# Risk factors for delay of adjuvant chemotherapy in non-metastatic breast cancer patients: A systematic review and meta-analysis involving 186982 patients

**DOI:** 10.1371/journal.pone.0173862

**Published:** 2017-03-16

**Authors:** Xiaofang He, Fen Ye, Bingcheng Zhao, Hailin Tang, Jin Wang, Xiangsheng Xiao, Xiaoming Xie

**Affiliations:** 1 Department of Breast Oncology, Sun Yat-Sen University Cancer Center, State Key Laboratory of Oncology in South China, Collaborative Innovation Center for Cancer Medicine, Guangzhou, Guangdong, China; 2 Department of Anesthesiology, First Affiliated Hospital of Sun Yat-Sen University, Guangzhou, Guangdong, China; Sudbury Regional Hospital, CANADA

## Abstract

**Purpose:**

Delay performance of adjuvant chemotherapy (AC) after surgery has been presented to affect survival of breast cancer patients adversely, but the risk factors for delay in initiation remain controversial. Therefore, we conducted this systematic review of the literature and meta-analysis aiming at identifying the risk factors for delay of adjuvant chemotherapy (DAC) in non-metastatic breast cancer patients.

**Methods:**

The search was performed on PubMed, Embase, Chinese National Knowledge Infrastructure and Wanfang Database from inception up to July 2016. DAC was defined as receiving AC beyond 8-week after surgery. Data were combined and analyzed using random-effects model or fixed-effects model for risk factors considered by at least 3 studies. Heterogeneity was analyzed with meta-regression analysis of year of publication and sample size. Publication bias was studied with Egger’s test.

**Results:**

A total of 12 observational studies including 186982 non-metastatic breast cancer patients were eligible and 12 risk factors were analyzed. Combined results demonstrated that black race (vs white; OR, 1.18; 95% CI, 1.01–1.39), rural residents (vs urban; OR, 1.60; 95% CI, 1.27–2.03) and receiving mastectomy (vs breast conserving surgery; OR, 1.35; 95% CI, 1.00–1.83) were significantly associated with DAC, while married patients (vs single; OR, 0.58; 95% CI, 0.38–0.89) was less likely to have a delay in initiation. No significant impact from year of publication or sample size on the heterogeneity across studies was found, and no potential publication bias existed among the included studies.

**Conclusions:**

Risk factors associated with DAC included black race, rural residents, receiving mastectomy and single status. Identifying of these risk factors could further help decisions making in clinical practice.

## Introduction

Breast cancer is the most common type of malignant tumor and second leading cause of death in women worldwide. It is estimated that there will be 249,260 new cases and 40,890 deaths in United States in 2016 [1], which places a heavy burden on the healthcare system. Surgery is the “gold standard” treatment for early breast cancer [2] and adjuvant chemotherapy (AC) has been proved to have a significant survival benefit [3]. Although the appropriate time interval from surgery to the start of AC has not been defined, many studies demonstrated that shorter time interval was associated with better survival outcomes [4–7]. A more recent meta-analysis reported that a 4-week increase in time to initiation of AC led to a significant increase in the risk of death [8]. The initiation of AC was regularly suggested within 8 to 12 weeks after surgery [9].

While worse survival outcome from delay of adjuvant chemotherapy (DAC) has been well established, the risk factors for DAC remain unknown. Since the risk factors could not be evaluated by randomized controlled trails, evidence from numerous observational studies demonstrated that the risk factors associated with DAC included demographics, clinical characteristics, pathologic characteristics and surgical approaches[5–7, 10–18]. However, their impact on DAC remain inconsistent.

Due to a lack of understanding of the risk factors, we therefore conducted this systematic review and meta-analysis to identify the impact of risk factors on DAC.

## Materials and methods

### Search strategy

A systematic review was conducted to identify all studies concerning the risk factors for DAC in non-metastatic breast cancer patients by searching PubMed, Embase, Chinese National Knowledge, and Wanfang Database from inception up to July 2016. Two investigators (XFH and BCZ) independently carried out the search using the following keywords simultaneously: (1) breast cancer or breast carcinoma or breast neoplasm or breast tumor; (2) adjuvant treatment or adjuvant chemotherapy; (3) delay chemotherapy or delayed chemotherapy. The reference lists of the selected articles were also reviewed for additional relevant studies.

### Eligibility criteria

The inclusion criteria were as follow: the time interval between surgery and administration of AC was defined; at least one risk factor concerning DAC was investigated; odds ratio (OR) or risk ratio (RR), and associated 95% confidence intervals (CI) were available or could be calculated from the original articles. Only full-report in English was included. For duplicated cases, the most comprehensive one was eligible for inclusion. Articles were excluded if they did not meet the above criteria, or the information provided was insufficient for the outcome data extraction or quality assessment.

### Data extraction

All the searched articles were independently reviewed by two investigators (XFH and BCZ). After reading the titles and abstracts, the full texts were retrieved for those potentially included articles to achieve further assessment for inclusion. Discordance in selection was solved through discussion. For the included studies, following data were extracted: author details, year of publication, data source if available, study location, sample size, age of participants, TNM stage, AC regimens if available, cut-off categorical value of time interval, any information about quality assessment under the guideline of the Newcastle-Ottawa Scale, any risk factor investigated, OR, RR and associated 95% CIs. The accuracy of extracted data was ultimately confirmed by a third investigator (FY).

### Statistical analysis

Data were combined and analyzed when the risk factor was adequately considered by at least 3 studies. Because all the included studies were observational, multivariate estimates were preferentially used. If not available, univariate estimates were extracted. When the OR, RR and associated 95%CIs were not present in the original article, we calculated OR by assessing the total number of events and total number of patients in each group. The 8-week delay was determined as the cut-off time point. For studies having different time points, the closest one to the 8-week was used. We measured the inter-study heterogeneity by using I^2^ statistic. Substantial heterogeneity was defined if an I^2^ value exceeded 50%. Forest plots were carried out to estimate the pooled ORs using the random-effects model when I^2^ value exceeded 50%, or the fixed-effects model when I^2^ value not exceeding 50%. Meta-regression analysis was performed to assess the impact of year of publication and sample size on the effect on the inter-study heterogeneity. The publication bias was assessed by Egger’s test. A two-tailed *p*-value < 0.05 was considered statistically significant. All the statistical analyses were conducted by Stata software (Stata SE 12.0). This systematic review and meta-analysis was performed under the guidelines of MOOSE [19].

## Results

### Study selection

**Fig 1:**
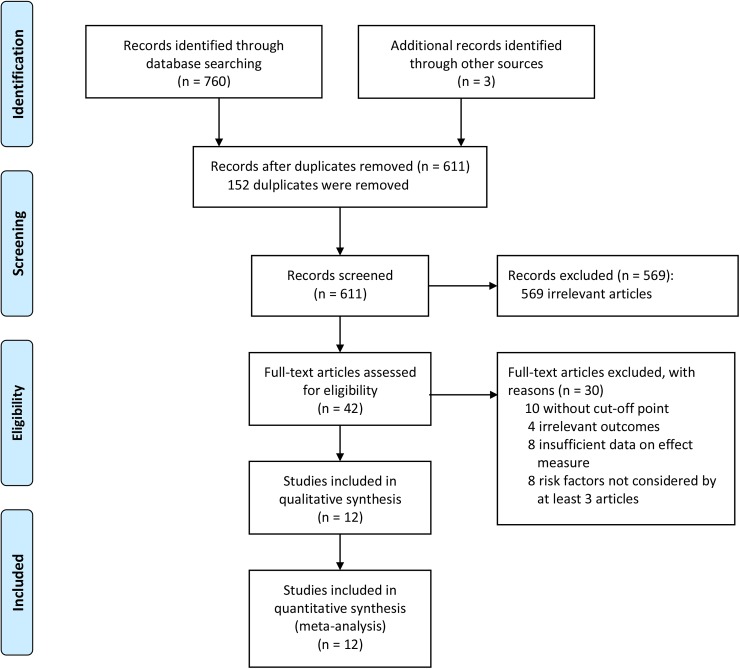
Flowchart of search and selection process for eligible studies.

The search and selection process for eligible studies was shown in Fig 1. A total of 760 potentially relevant articles were identified, and 3 additional articles were included by manually screening the reference lists. 152 duplicates were found and removed. After reading the titles and abstracts, 569 irrelevant studies were excluded and the remaining 42 articles were reviewed in full text. Of these, 30 studies were excluded because of various reasons. Ultimately, a total of 12 articles were included for meta-analysis after detailed assessments [5–7, 10–18].

### Study characteristics

Table 1 summarized the characteristics of the eligible studies. A total of 186982 patients with stage I, II, or III breast cancer were encompassed between 2006 and 2014. The cutoff values of DAC were from 45 to 90 days. Most of the studies were carried out in United States, with one in New Zeeland and one in Canada. No prospective studies were included.

**Table 1:** Main characteristics of included studies.

Study	Location	Data source	No. of patients	Age/years	Stage	Chemotherapy regimen ^a^	Delay cutoff
Hershman, 2006 ^7^	United States	SEER	5007	>65	Ⅰ, Ⅱ	N/S	3 months
Lohrisch, 2006 ^5^	Canada	Breast Cancer Outcomes Unit Database of the British Columbia Cancer Agency	2594	47(median)	Ⅰ, Ⅱ	AC, CEF, FAC/CAF, CMF	12weeks
Jara Sanchez, 2007 ^10^	United States	El A´ lamo	2782	21–93	Ⅰ, Ⅱ, Ⅲ	CMF, A-based, T-based, TA	9 weeks
Alderman, 2010 ^11^	United States	N/S	3643	N/S	Ⅰ, Ⅱ, Ⅲ	N/S	8weeks
Fedewa, 2010 ^12^	United States	National Cancer Data Base	107587	18–99	Ⅰ, Ⅱ, Ⅲ	N/S	90 days
Balasubramanian, 2012 ^13^	United States	New Jersey State Medicaid Files	365	20–64	Ⅰ, Ⅱ, ⅢA	CAF-based	3 months
Simon, 2012 ^14^	United States	Henry Ford Health System	2234	61.2 (average)	Ⅰ, Ⅱ, Ⅲ	N/S	60 days
Freedman, 2013 ^15^	United States	SEER	54592	≥66	Ⅰ, Ⅱ, Ⅲ	N/S	90 days
Sheppard, 2013 ^16^	United States	N/S	359	25–89	N/S	N/S	90 days
Barry, 2014 ^17^	United States	N/S	70	30–65	Ⅰ, Ⅱ	N/S	45 days
Gagliato Dde, 2014 ^6^	United States	Breast Medical Oncology Institutional Database	6827	19–85	Ⅰ, Ⅱ, Ⅲ	A-based, TA-based, or other type.	60 days
Seneviratne, 2014 ^18^	New Zealand	Waikato breast cancer register	922	N/S	Ⅰ, Ⅱ, Ⅲ	N/S	60 days

Abbreviation: SEER: Surveillance, Epidemiology, and End Results Program; N/S: not stated.

^a^ AC = doxorubicin + cyclophosphamide; CEF = cyclophosphamide + epirubicin + fluorouracil; FAC/CAF = fluorouracil + doxorubicin + cyclophosphamide; CMF = cyclophosphamide + methotrexate + fluorouracil; A-based = anthracycline-based; T-based = taxane-based; TA = anthracycline + taxane; CAF-based = cyclophosphamide, doxorubicin/ epirubicin, 5-fluorouracil, or a combination of these agents.

### Quality assessment

To assess the quality of the observational studies, selection of participants, study comparability, and ascertainment of exposure were examined for all the included studies based on the Newcastle-Ottawa Scale [20] (shown in Table 2). A maximum of 9 starts could be obtained as the highest quality. The scores assessed for the eligible studies were ranged from 6 to 9, all of which were identified as very good or good in quality [21].

**Table 2:** Methodological quality of studies included in the meta-analysis.

Study	Case definition adequate	Representativeness of the cases	Selection of controls	Definition of controls	Control for important factors ^a^	Ascertainment of exposure	Same method of ascertainment for cases and controls	Non-response rate	Total quality scores
Hershman, 2006 ^7^	☆	☆	☆	☆	☆☆	☆	☆	☆	9
Lohrisch, 2006 ^5^	☆	—	—	☆	☆	☆	☆	☆	6
Jara Sanchez, 2007 ^10^	☆	☆	☆	☆	☆	☆	☆	☆	8
Alderman, 2010 ^11^	☆	☆	☆	☆	☆	☆	☆	☆	8
Fedewa, 2010 ^12^	☆	☆	☆	☆	☆☆	☆	☆	☆	9
Balasubramanian, 2012 ^13^	☆	☆	☆	☆	☆	☆	☆	☆	8
Simon, 2012 ^14^	☆	☆	☆	☆	☆☆	☆	☆	☆	9
Freedman, 2013 ^15^	☆	☆	☆	☆	☆☆	☆	☆	☆	9
Sheppard, 2013 ^16^	☆	—	—	☆	☆	☆	☆	☆	6
Barry, 2014 ^17^	☆	—	—	☆	☆☆	☆	☆	☆	7
Gagliato Dde, 2014 ^6^	☆	—	—	☆	☆☆	☆	☆	☆	7
Seneviratne, 2014 ^18^	☆	☆	☆	☆	☆	☆	☆	☆	8

^a^ A maximum of 2 stars could be awarded for this item. Studies that controlled for age received one star, whereas studies that controlled for other factors received an additional star.

### Risk factors extracted for meta-analysis

In total, 12 risk factors were extracted from the included studies, including age at diagnosis (<70 vs ≥70 years), race (white vs black), county (urban vs rural), comorbidity status (Charlson score 0 vs ≥1), marital status (single vs married), TNM stage (I + II vs III), hormone receptor status (estrogen receptor [ER] and progesterone receptor [PR] negative vs ER and/or PR positive), histological grade (well and/or moderately differentiated vs poorly differentiated), surgical approach (breast conserving surgery [BCS]vs mastectomy), number of involved nodes (0–9 vs ≥ 10), tumor size (0-5cm vs > 5cm), and lymphatic vascular invasion status (absent vs present).

### Risk factors contributing to DAC

**Fig 2:**
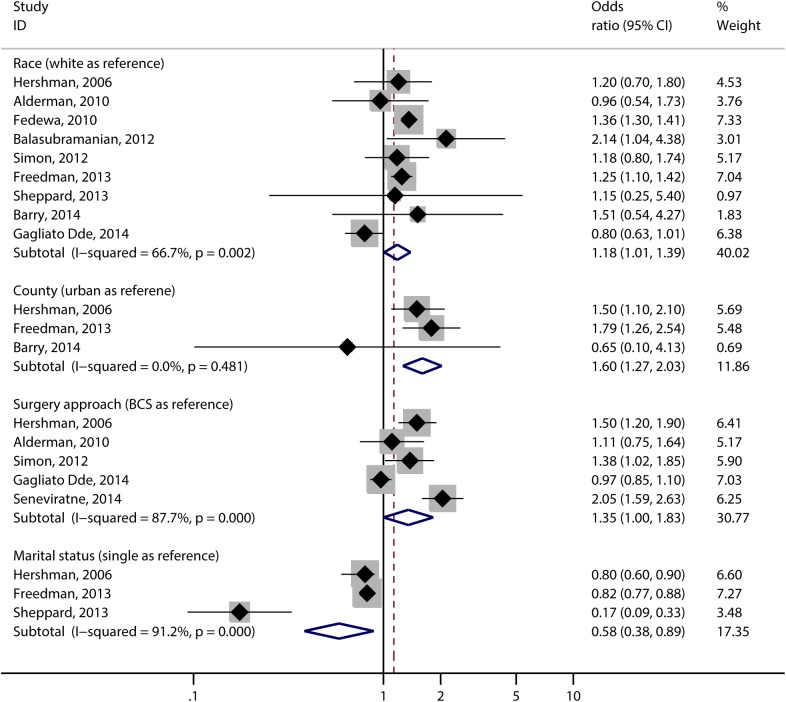
Forrest plots of risk factors that were contributed to DAC.

The pooled results demonstrated that an 18% increased risk of DAC for black race compared with white race (OR, 1.18; 95% CI, 1.01–1.39; I^2^ = 66.7%), a 60% higher risk for rural residents than urban residents (OR, 1.60; 95% CI, 1.27–2.03; I^2^ = 0.0%), and a 35% higher risk for patients receiving mastectomy than patients receiving BCS (OR, 1.35; 95% CI, 1.00–1.83; I^2^ = 87.7%). While married patients were less likely to have a delay in initiation compared with single patients (OR, 0.58; 95% CI, 0.38–0.89; I^2^ = 91.2%, Fig 2).

### Risk factors not contributing to DAC

**Fig 3:**
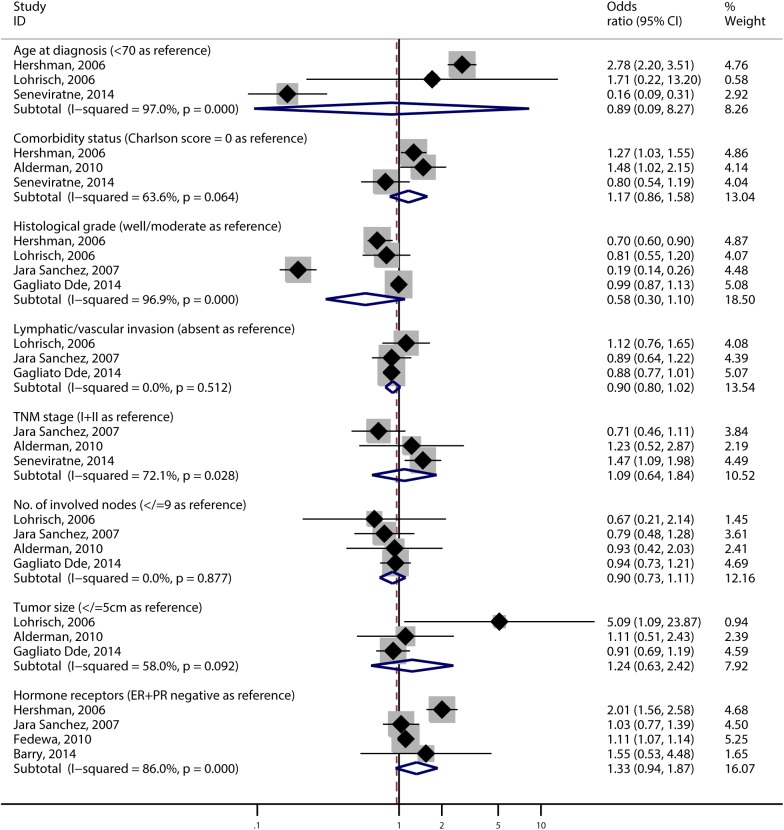
Forrest plots of risk factors that were not contributed to DAC.

Older than 70 years (OR, 0.89; 95% CI, 0.09–8.27; I^2^ = 97.2%), worse comorbidity status (OR, 1.17; 95% CI, 0.86–1.58; I^2^ = 63.6%), poorer histological differentiation (OR, 0.58; 95% CI, 0.30–1.10; I^2^ = 96.9%), presence of lymphatic vascular invasion (OR, 0.90; 95% CI, 0.80–1.02; I^2^ = 0.0%), higher TNM stage (OR, 1.09; 95% CI, 0.64–1.84; I^2^ = 72.1%), involved nodes ≥10 (OR, 0.90; 95% CI, 0.73–1.11; I^2^ = 0.0%), tumor size > 5cm (OR, 1.24; 95% CI, 0.63–2.42; I^2^ = 58.0%) and ER / PR positive status (OR, 1.33; 95% CI, 0.94–1.87; I^2^ = 86.0%, Fig 3) were not correlated with an increased risk of DAC.

### Meta-regression analysis and publication bias assessment

Meta-regression analysis suggested that year of publication and sample size did not have a significant impact on the heterogeneity across studies for each factor. Egger’s test demonstrated that no potential publication bias existed among the included studies for various factors (shown in Table 3).

**Table 3:** Meta-regression analysis and Egger’s test for various factors.

	No. of studies	Meta-regression analysis ^a^		Egger’ s test (*P* value)
		Year of publication (*P* value)	Sample size (*P* value)	
Race	9	0.546	0.389	0.283
County	3	0.914	0.549	0.455
Surgical approach	5	0.615	0.156	0.257
Marital status	3	0.685	0.616	0.372
Age	3	0.078	0.159	0.599
Comorbidity status	3	0.504	0.396	0.738
Histological grade	4	0.892	0.669	0.555
Lymphatic vascular invasion	3	0.587	0.605	0.311
TNM stage	3	0.231	0.593	0.661
No. of involved nodes	4	0.766	0.824	0.277
Tumor size	3	0.346	0.484	0.288
Hormone receptors	4	0.945	0.740	0.439

^a^ Adjustment for both year of publication and sample size were performed when number of studies were at least 4.

## Discussion

In this meta-analysis, data on 186982 non-metastatic breast cancer patients from 12 studies were analyzed in characterizing the risk factors related to DAC. Combined results demonstrated that black race, rural residents and receiving mastectomy had significantly higher likelihood of experiencing DAC, while married patients were at lower risk. To the best of our knowledge, this is the first systematic review and meta-analysis evaluating the previously reported risk factors associated with DAC.

Results from 9 subset studies of our meta-analysis suggested that black race was associated with an 18% increased risk of DAC compared with white race, which was consistent with the conclusions of previous studies [22, 23]. However, the pooled result should be interpreted cautiously because the magnitude of race disparity on DAC was quite modest (18%) and high heterogeneity of 66.7% was observed across studies. African American women were the major component of black race in our study. The reasons for them to have a higher risk of DAC might result from following aspects: low education level, disadvantaged socioeconomic status (SES), unavailability of transportation and a lack of insurance [24–27]. Since the disparity of SES between black and white race would affect their decision on the initiation of AC after surgery [28], hence, we further divided these 9 studies into two groups: SES unknown between black and white race (U-SES), and lower SES for black race than white race (L-SES). The meta-analysis for these two groups (shown in S1 Fig) demonstrated that black race in L-SES group had a 35% increased risk of DAC, which was higher than the pooled result (18% increased risk) of the 9 studies, while there was no significant difference in U-SES group. This could partially explain that the lower SES of black race might push them to start AC administration later than white race. More work is warranted to further address this issue.

In addition, our combined result from 4 studies demonstrated that mastectomy was associated with an 83% increased risk of DAC compared with BCS. Because the extent of mastectomy is larger than that of BCS, it is more likely for patients receiving mastectomy to suffer greater complications, including surgical site infections, wound dehiscence and skin flap necrosis [29, 30], which could result in a longer recovery period and so delaying AC administration. A recent meta-analysis suggested that mastectomy with immediate breast reconstruction did not necessarily delay the initiation of AC compared with mastectomy only [31]. However, our study did not analyze the effect of mastectomy with immediate breast reconstruction on DAC, because no sufficient data could be extracted from the included studies. Therefore, more future studies evaluating mastectomy with immediate breast reconstruction and BCS on impact of DAC are warranted to further address this issue.

Besides, three studies of the current meta-analysis documented DAC in rural residents, which was consistent with the previously reported studies and the reasons has been well interpreted that rural residents had less access to comprehensive hospitals and difficult transportation to the long-distant qualified hospitals [14, 24]. Otherwise, we also observed that married patients were 42% less likely to delay the AC than single patients, since married patients usually gain more support from family members to accept clinician’s recommendation and start treatment [32, 33]. It is noted that several risk factors evaluated in our meta-analysis did not have significant association with DAC as mentioned in the results, which might be attributed to few studies included and inconsistent findings across included studies.

The greatest strengths of the current study are the large sample sizes of over 180000 non-metastatic breast cancer patients and wide range of evaluated risk factors. The study indicated that black race, receiving mastectomy, rural residents and single status were significantly associated with DAC, which could be helpful for clinicians to identify the specific population groups and to start AC early. Furthermore, our work would promote the health system to pay extra attention to improve the medical conditions for patients at increased risk of delay of treatment. Of note, our meta-analysis did not focus on the survival outcomes caused by DAC. One reason is that we could not extract sufficient data from the eligible studies, since there were only 4 included studies referring to the survival outcomes. Another reason is that many previous studies and meta-analysis have demonstrated that longer time interval was associated with worse survival outcomes. Nevertheless, we did not deny that in some cases, DAC was not associated with increased risk of mortality, such as in a cohort of postmenopausal, ER-positive breast cancer patients following adjuvant endocrine therapy [34, 35].

Several potential limitations of our meta-analysis should be considered. First, data were extracted from observational studies, so the inherent potential bias caused by unmeasured and uncontrolled confounders were inevitable. Second, high heterogeneity across studies was identified, although meta-regression analysis was performed to estimate the impact of year of publication and sample size and no statistically significant result was found. Thus, the interpretation of our results should be with caution. Besides, the cutoff time point of DAC was not uniform among the eligible studies, ranging from 45 to 90days, which might probably result in variability across studies and so could distort our findings.

In conclusion, our meta-analysis of the current literature demonstrated that black race, rural residents, receiving mastectomy and single status led to significantly increased risk of experiencing DAC in non-metastatic breast cancer patients. Identification of these factors could be helpful for personalized treatment planning.

## Supporting information

S1 Fig Forrest plots of race stratified by socioeconomic status. (TIF)

S1 Table PRISMA Checklist. (DOC)

S2 Table Specific data of odds ratios for each risk factor by studies. (DOC)

S3 Table Assessment criteria of socioeconomic status of included studies. (DOC)
