# Supplementary figures and images for: Risk factors for delay of adjuvant chemotherapy in non-metastatic breast cancer patients: A systematic review and meta-analysis involving 186982 patients
Source: PLoS One. 2017 Mar 16;12(3):e0173862. doi: 10.1371/journal.pone.0173862 (PMC5354309; doi:10.1371/journal.pone.0173862)

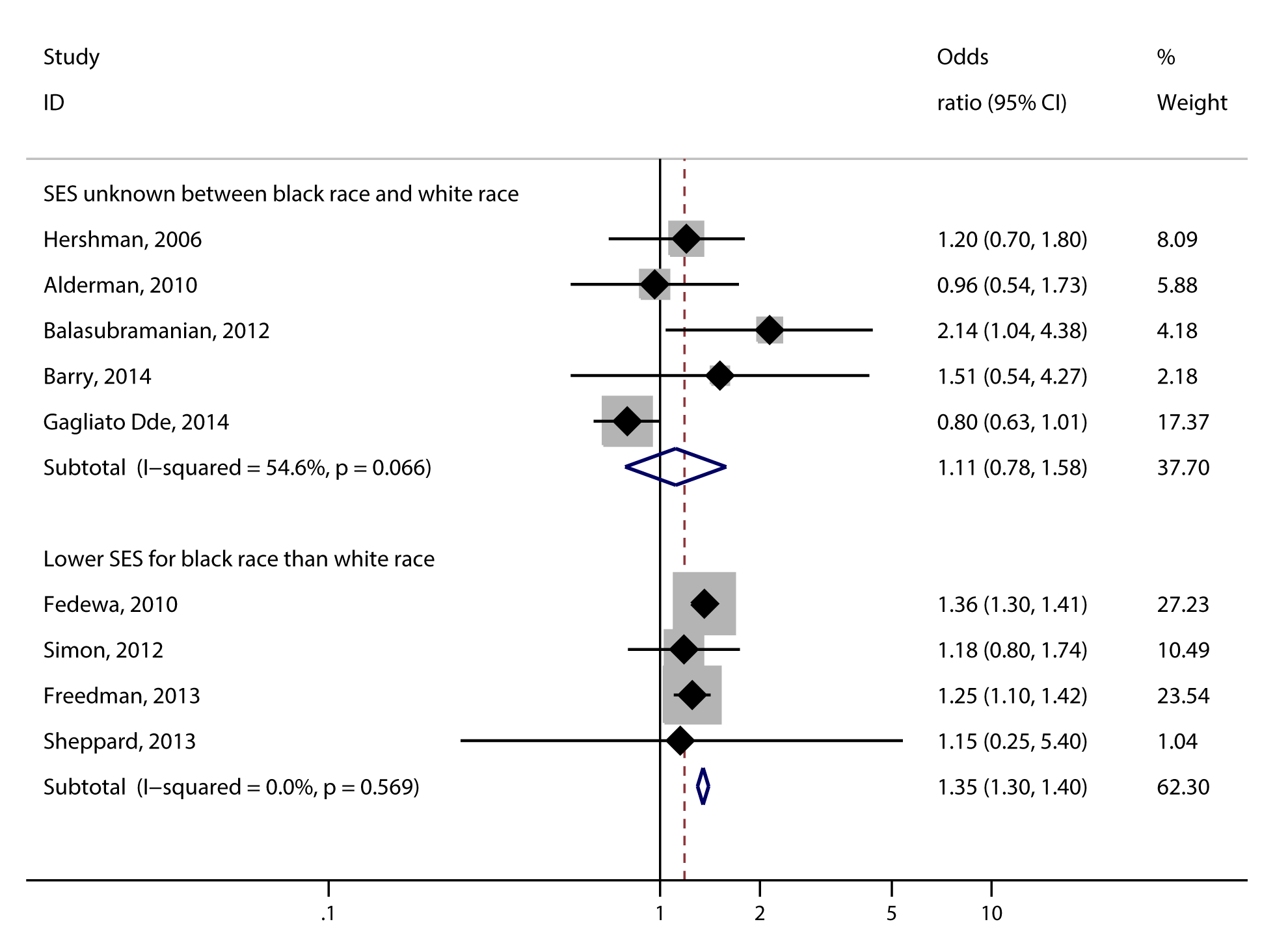

Supplement: S1 Fig — (TIF) [file pone.0173862.s001.tif]
