# Supplementary material for: Risk factors for delay of adjuvant chemotherapy in non-metastatic breast cancer patients: A systematic review and meta-analysis involving 186982 patients
Source: PLoS One. 2017 Mar 16;12(3):e0173862. doi: 10.1371/journal.pone.0173862 (PMC5354309; doi:10.1371/journal.pone.0173862)
Supplement: S2 Table — (DOC) [file pone.0173862.s003.doc]

**S2. Specific data of odds ratios for each risk factor by studies.**

|  | **No. of patients** | **No. of patients(delay)** | **OR** | **95% CI** |
| --- | --- | --- | --- | --- |
| **Race** |  |  |  |  |
| Hershman, 2006 |  |  |  |  |
| White | 3980 | 417 | 1 |  |
| Black | 289 | 33 | 1.2 | 0.7-1.8 |
| Alderman, 2010 |  |  |  |  |
| White | 2190 | 168 | 1 |  |
| Black | 176 | 13 | 0.963 | 0.537-1.728 |
| Fedewa, 2010 * |  |  |  |  |
| White |  |  | 1 |  |
| Black |  |  | 1.36 | 1.30-1.41 |
| Balasubramanian, 2012 |  |  |  |  |
| White | 215 | 22 | 1 |  |
| Black | 107 | 21 | 2.14 | 1.04-4.38 |
| Simon, 2012 |  |  |  |  |
| White | 274 | 221 | 1 |  |
| Black | 131 | 177 | 1.18 | 0.80-1.74 |
| Freedman, 2013 * |  |  |  |  |
| White |  |  | 1 |  |
| Black |  |  | 1.25 | 1.10-1.42 |
| Sheppard, 2013 |  |  |  |  |
| White | 137 | 12 | 1 |  |
| Black | 153 | 57 | 1.15 | 0.25-5.40 |
| Barry, 2014 |  |  |  |  |
| White | 25 | 22 | 1 |  |
| Black | 9 | 12 | 1.515 | 0.537-4.274 |
| Gagliato Dde, 2014 |  |  |  |  |
| White | 4863 | 976 | 1 |  |
| Black | 557 | 89 | 0.796 | 0.630-1.006 |
| **County** |  |  |  |  |
| Hershman, 2006 |  |  |  |  |
| Urban | 4069 | 397 | 1 |  |
| Rural | 461 | 80 | 1.5 | 1.1-2.1 |
| Freedman, 2013 * |  |  |  |  |
| Urban |  |  | 1 |  |
| Rural |  |  | 1.79 | 1.26-2.54 |
| Barry, 2014 |  |  |  |  |
| Urban | 32 | 33 | 1 |  |
| Rural | 3 | 2 | 0.646 | 0.101-4.128 |
| **Surgery approach** |  |  |  |  |
| Hershman, 2006 |  |  |  |  |
| Breast conserving surgery | 2278 | 160 | 1 |  |
| Mastectomy | 2252 | 317 | 1.5 | 1.2-1.9 |
| Alderman, 2010 |  |  |  |  |
| Breast conserving surgery | 1387 | 91 | 1 |  |
| Mastectomy | 536 | 39 | 1.109 | 0.752-1.635 |
| Simon, 2012 * |  |  |  |  |
| Breast conserving surgery |  |  | 1 |  |
| Mastectomy |  |  | 1.38 | 1.02-1.85 |
| Gagliato Dde, 2014 |  |  |  |  |
| Breast conserving surgery | 2537 | 505 | 1 |  |
| Mastectomy | 3172 | 612 | 0.969 | 0.852-1.103 |
| Seneviratne, 2014 |  |  |  |  |
| Breast conserving surgery | 1153 | 165 | 1 |  |
| Mastectomy | 464 | 136 | 2.048 | 1.593-2.633 |
| **Marital status** |  |  |  |  |
| Hershman, 2006 |  |  |  |  |
| Single | 2169 | 278 | 1 |  |
| Married | 2361 | 199 | 0.8 | 0.6-0.9 |
| Freedman, 2013 * |  |  |  |  |
| Single |  |  | 1 |  |
| Married |  |  | 0.82 | 0.77-0.88 |
| Sheppard, 2013 |  |  |  |  |
| Single | 120 | 57 | 1 |  |
| Married | 168 | 14 | 0.175 | 0.093-0.329 |
| **Age at diagnosis** |  |  |  |  |
| Hershman, 2006 |  |  |  |  |
| <70 | 1852 | 95 | 1 |  |
| ≥70 | 2678 | 382 | 2.781 | 2.204-3.508 |
| Lohrisch, 2006 |  |  |  |  |
| <70 | 2469 | 111 | 1 |  |
| ≥70 | 13 | 1 | 1.711 | 0.222-13.196 |
| Seneviratne, 2014 |  |  |  |  |
| <70 | 1336 | 291 | 1 |  |
| ≥70 | 281 | 10 | 0.163 | 0.086-0.311 |
| **Comorbidity score** |  |  |  |  |
| Hershman, 2006 |  |  |  |  |
| 0 | 3338 | 328 | 1 |  |
| ≥1 | 1189 | 148 | 1.267 | 1.032-1.555 |
| Alderman, 2010 |  |  |  |  |
| 0 | 2091 | 148 | 1 |  |
| ≥1 | 363 | 38 | 1.479 | 1.018-2.149 |
| Seneviratne, 2014 |  |  |  |  |
| 0 | 1408 | 269 | 1 |  |
| ≥1 | 209 | 32 | 0.801 | 0.540-1.189 |
| **Histological grade** |  |  |  |  |
| Hershman, 2006 |  |  |  |  |
| Well/Moderately differentiated | 1715 | 231 | 1 |  |
| Poorly differentiate | 2088 | 151 | 0.7 | 0.6-0.9 |
| Lohrisch, 2006 |  |  |  |  |
| Well/Moderately differentiated | 1033 | 51 | 1 |  |
| Poorly differentiate | 1392 | 56 | 0.815 | 0.553-1.201 |
| Jara Sanchez, 2007 |  |  |  |  |
| Well/Moderately differentiated | 1727 | 161 | 1 |  |
| Poorly differentiate | 829 | 65 | 0.194 | 0.144-0.262 |
| Gagliato Dde, 2014 |  |  |  |  |
| Well/Moderately differentiated | 2369 | 462 | 1 |  |
| Poorly differentiate | 3076 | 595 | 0.992 | 0.869-1.133 |
| **Lymphatic/vascular invasion** |  |  |  |  |
| Lohrisch, 2006 |  |  |  |  |
| Absent | 1330 | 57 | 1 |  |
| Present | 1061 | 51 | 1.122 | 0.762-1.650 |
| Jara Sanchez, 2007 |  |  |  |  |
| Absent | 1887 | 172 | 1 |  |
| Present | 669 | 54 | 0.886 | 0.644-1.217 |
| Gagliato Dde, 2014 |  |  |  |  |
| Absent | 3822 | 778 | 1 |  |
| Present | 1888 | 339 | 0.882 | 0.768-1.014 |
| **TNM stage** |  |  |  |  |
| Jara Sanchez, 2007 |  |  |  |  |
| Ⅰ+ Ⅱ | 2191 | 202 | 1 |  |
| Ⅲ | 364 | 24 | 0.715 | 0.462-1.108 |
| Alderman, 2010 |  |  |  |  |
| Ⅰ+ Ⅱ | 2389 | 180 | 1 |  |
| Ⅲ | 65 | 6 | 1.225 | 0.524-2.866 |
| Seneviratne, 2014 |  |  |  |  |
| Ⅰ+ Ⅱ | 1341 | 231 | 1 |  |
| Ⅲ | 276 | 70 | 1.472 | 1.094-1.982 |
| **No. of involved nodes** |  |  |  |  |
| Lohrisch, 2006 |  |  |  |  |
| 0-9 | 2381 | 109 | 1 |  |
| ≥10 | 98 | 3 | 0.669 | 0.209-2.143 |
| Jara Sanchez, 2007 |  |  |  |  |
| 0-9 | 2289 | 207 | 1 |  |
| ≥10 | 267 | 19 | 0.787 | 0.484-1.280 |
| Alderman, 2010 |  |  |  |  |
| 0-9 | 2351 | 179 | 1 |  |
| ≥10 | 99 | 7 | 0.929 | 0.425-2.029 |
| Gagliato Dde, 2014 |  |  |  |  |
| 0-9 | 5196 | 1019 | 1 |  |
| ≥10 | 427 | 79 | 0.943 | 0.735-1.210 |
| **Tumor size** |  |  |  |  |
| Lohrisch, 2006 |  |  |  |  |
| ≤5 | 2453 | 107 | 1 |  |
| ＞5 | 9 | 2 | 5.094 | 1.087-23.867 |
| Alderman, 2010 |  |  |  |  |
| ≤5 | 2329 | 175 | 1 |  |
| ＞5 | 84 | 7 | 1.109 | 0.505-2.434 |
| Gagliato Dde, 2014 |  |  |  |  |
| ≤5 | 5206 | 1031 | 1 |  |
| ＞5 | 356 | 64 | 0.908 | 0.690-1.194 |
| **Hormone receptors** |  |  |  |  |
| Hershman, 2006 |  |  |  |  |
| ER and PR negative | 1307 | 81 | 1 |  |
| ER and/or PR positive | 2581 | 321 | 2.007 | 1.559-2.584 |
| Jara Sanchez, 2007 |  |  |  |  |
| ER and PR negative | 798 | 69 | 1 |  |
| ER and/or PR positive | 1758 | 157 | 1.033 | 0.769-1.388 |
| Fedewa, 2010 * |  |  |  |  |
| ER and PR negative |  |  | 1 |  |
| ER and/or PR positive |  |  | 1.11 | 1.07-1.14 |
| Barry, 2014 |  |  |  |  |
| ER and PR negative | 11 | 8 | 1 |  |
| ER and/or PR positive | 24 | 27 | 1.547 | 0.534-4.482 |

Abbreviations: OR: odds ratio; CI: confidence interval; ER: estrogen receptor; PR: progesterone receptor.

* No. of patients in each group was unavailable in these articles.
