# Supplementary material for: Risk factors for delay of adjuvant chemotherapy in non-metastatic breast cancer patients: A systematic review and meta-analysis involving 186982 patients
Source: PLoS One. 2017 Mar 16;12(3):e0173862. doi: 10.1371/journal.pone.0173862 (PMC5354309; doi:10.1371/journal.pone.0173862)
Supplement: S3 Table — (DOC) [file pone.0173862.s004.doc]

**S3. Assessment criteria of socioeconomic status** of included studies

| **Study** | **Assessment criteria of socioeconomic status** |
| --- | --- |
| Hershman, 2006 ^7^ | A socioeconomic status score generated from a hierarchy of income in quartiles including median income in the census tract of residence, median income in the zip code of residence, census tract per capita income, and zip code per capita income. |
| Lohrisch, 2006 ^5^ | N/S |
| Jara Sanchez, 2007 ^10^ | N/S |
| Alderman, 2010 ^11^ | Median household income: Q1: $0 to <$35,426; Q2: $35,426 to <$44,639; Q3: $44,639 to <$58,844; Q4: $58,844-$159,538; unknown. |
| Fedewa, 2010 ^12^ | Proportion of patients without high school diploma and primary payer and/or insurance type grouped into the following categories: Medicaid, Medicare (including Medicare alone and with supplement), uninsured (which includes not insured, charity write-off, and self-pay), private insurance plans (health maintenance organizations, preferred provider organizations), and other/unknown. |
| Balasubramanian, 2012 ^13^ | N/S |
| Simon, 2012 ^14^ | A deprivation index in quartiles that captures the economic and social conditions of neighborhoods including unemployment, poverty, overcrowding, telephone, and automobile availability. |
| Freedman, 2013 ^15^ | High school diploma rates and median income in quartiles. |
| Sheppard, 2013 ^16^ | Education level: no college education; some college; bachelors and above. |
| Barry, 2014 ^17^ | Insurance status: government, private, none. |
| Gagliato Dde, 2014 ^6^ | N/S |
| Seneviratne, 2014 ^18^ | Deprivation score according to the New Zealand Deprivation Index 2006 (NZDep06) on a scale from 1 to 10. |

Abbreviation: N/S: not stated.
